# Macropinocytic entry of isolated mitochondria in epidermal growth factor-activated human osteosarcoma cells

**DOI:** 10.1038/s41598-017-13227-0

**Published:** 2017-10-10

**Authors:** Dipali Patel, Joanna Rorbach, Kate Downes, Maciej J. Szukszto, Marcin L. Pekalski, Michal Minczuk

**Affiliations:** 1 MRC Mitochondrial Biology Unit, University of Cambridge, Hills Road, Cambridge, CB2 0XY UK; 2 CIMR, University of Cambridge, Hills Road, Cambridge, CB2 0XY UK; 3 Present Address: Department of Medical Biochemistry and Biophysics, Karolinska Institutet, Solna, Sweden; 4 Present Address: Department of Haematology, University of Cambridge, NHS Blood and Transplant, Long Road, Cambridge, CB2 0PT UK

## Abstract

Mammalian mitochondria can be transferred between cells both in culture and *in vivo*. There is evidence that isolated mitochondria enter cells by endocytosis, but the mechanism has not been fully characterised. We investigated the entry mechanism of isolated mitochondria into human osteosarcoma (HOS) cells. Initially we confirmed that respiratory-competent cells can be produced following incubation of HOS cells lacking mitochondrial DNA (mtDNA) with functional exogenous mitochondria and selection in a restrictive medium. Treatment of HOS cells with inhibitors of different endocytic pathways suggest that uptake of EGFP-labelled mitochondria occurs via an actin-dependent endocytic pathway which is consistent with macropinocytosis. We later utilised time-lapse microscopy to show that internalised mitochondria were found in large, motile cellular vesicles. Finally, we used confocal imaging to show that EGFP-labelled mitochondria colocalise with a macropinocytic cargo molecule during internalisation, HOS cells produce membrane ruffles interacting with external mitochondria during uptake and EGFP-labelled mitochondria are found within early macropinosomes inside cells. In conclusion our results are consistent with isolated mitochondria being internalised by macropinocytosis in HOS cells.

## Introduction

Mitochondria are double membrane bound organelles involved in diverse cellular processes, which include producing ATP, cellular Ca^2+^ homeostasis, autophagy, apoptosis and haeme biosynthesis. In addition, human mitochondria carry multiple copies of a double-stranded, circular DNA genome that is 16.5 kb in size. This encodes 13 essential subunits of the electron transport chain and ATP synthase, allowing cells to carry out oxidative phosphorylation (OxPhos), 22 tRNAs and 2 ribosomal RNAs^1^. Mutations in either mitochondrial DNA (mtDNA) or in nuclear-encoded mitochondrial proteins are a well-documented cause of human genetic disease^2^. Mitochondria display interesting characteristics as organelles, since they are dynamic structures existing as discrete units or undergoing fusion to form tubular networks. In addition, the last thirty years have provided evidence that mitochondria are mobile structures able to undergo transfer into cells.

Current data suggest that two forms of mitochondrial transfer (also referred to as “mitochondrial transformation”) take place in culture and *in vivo*: (i) an intercellular type occurring between cells^3^ and (ii) an extracellular type which transports mitochondria into cells from the environment^4^. Intercellular mitochondrial transfer seems to occur by two mechanisms either across nanotubes connecting cells^5,6^ or through the release and internalisation of vesicles^5,7,8^. Transfer of isolated mitochondria into mammalian cells is thought to be mediated by endocytosis.

Endocytosis is the production of new internal membranes from the plasma membrane leading to internalisation of membrane lipids, proteins and extracellular fluid into the cell. This process is important for the transport of material into cells. At least ten separate endocytic routes have been identified. All involve deformation of the plasma membrane to facilitate import of materials, but pathways vary in function, morphology and cargo. Clathrin-mediated endocytosis (CME) is defined as receptor-mediated uptake of extracellular material at the cell surface into clathrin-coated vesicles. Caveolae-mediated endocytosis involves internalisation of cargo into flask-shaped invaginations of the plasma membrane^9^. Both caveolae-mediated endocytosis and CME require dynamin, a mechanochemical enzyme, which mediates membrane fission during vesicle formation^10^. Macropinocytosis internalises extracellular fluid or particles into large vesicles that are between 0.2–5 μm in diameter. These vesicles are formed by actin-rich protrusions of the plasma membrane, called membrane ruffles^11^. These structures are variable in their morphology and include planar lamellipodia, circular dorsal ruffles (ring-shaped), finger-like filopodia and membrane blebs (large extrusions of the plasma membrane). Membrane ruffles can extend and fold over cargo or develop into cup-shaped structures that fuse over extracellular material. The ruffles form spontaneously or in response to growth factors, peptides and phorbol esters, which activates a receptor tyrosine-kinase pathway. This leads to the recruitment of multiple downstream effectors, that results in actin polymerisation, generation of membrane ruffles and macropinosome formation^12^.

Several studies have reported that isolated mitochondria enter mammalian cells by endocytosis^13–18^. However, there is disagreement regarding the underlying mechanism. Two groups have suggested that macropinocytosis mediates mitochondrial internalisation in a few human cell lines^16,18^. However, another study reported that mitochondrial internalisation in rat cardomyocytes is mediated by an actin-dependent endocytic pathway that is not macropinocytosis^17^. The existing studies provide conflicting and incomplete evidence regarding pathways mediating mitochondrial entry in mammalian cells. It is possible that there are species or cell specifc differences in the pathways responsible for mitochondrial entry. However, with the exception of recent analyses presented by Kesner *et al*., current evidence supporting the involvement of endocytic pathways in mitochondrial entry has relied almost exclusively on endocytic inhibitor data. Since these compounds can inhibit multiple endocytic pathways^19^, inhibitor data alone cannot be reliably used to investigate the routes of internalisation for an endocytic cargo.

Here, we confirm the previous observations on internalisation of exogenous mitochondria being able to restore OxPhos function to recipient human cells and that they can be stably maintained. Importantly, we present detailed evidence using endocytic inhibitors, endocytic agonists and confocal microscopy that mitochondrial entry into human osteosarcoma (HOS) cells occurs by macropinocytosis.

## Results

### Functional mitochondria restore OxPhos in ρ^0^ cells and are maintained in recipient human cells

**Figure 1:**
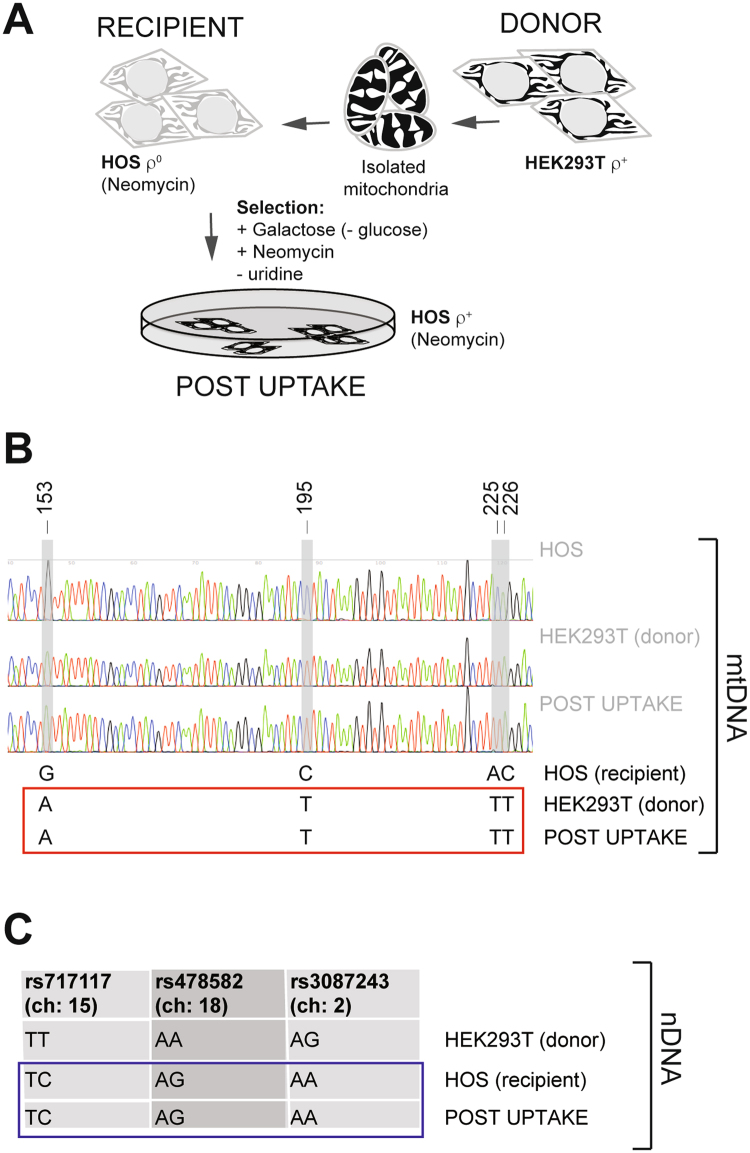
Isolated mitochondria complement defective oxidative phosphorylation in ρ^0^ cells and are stably maintained in recipient cells. (**A**) Schematic showing selection for recipient HOS cells that have imported mitochondria. (**B**) Mitochondrial DNA genotyping of ρ^0^ HOS cells, donor HEK293T cells and mitochondrial uptake clones. Four SNPs in the mtDNA non-coding region at positions 153, 195, 225 and 226 were used to distinguish between the recipient and donor cell mitochondria. Representative results of three independent analyses. (**C**) Nuclear DNA genotyping of HOS ρ^0^ cells, donor HEK293T cells and mitochondrial uptake clones. Three reference SNPs (refSNPs) were used to distinguish between the recipient and donor cell nDNA. HOS cells mtDNA reference sequence is given for the comparison with HEK293T mtDNA. Representative results of three independent analyses.

Firstly, we investigated whether purified mitochondria could be introduced into HOS ρ^0^ cells, which lack mtDNA as reported in previously published studies. Isolated ρ^+^ mitochondria from HEK293T cells were incubated with HOS ρ^0^ cells. Successful uptake of functional mitochondria by recipient cells should result in their gaining the ability to oxidatively phosphorylate. We used galactose medium lacking uridine and pyruvate to select recipient cells that could perform OxPhos. Additional drug selection was applied to prevent growth (or cell fusion) of any donor HEK293T cells, that might have survived the mitochondrial isolation procedure (Fig. 1A). An average of 9 respiratory competent clones per 1.5 × 10^5^ HOS ρ^0^ cells was recovered by this method using 125 µg/ml of isolated mitochondria in 3 independent experiments (Table 1). Clones were able to survive in OxPhos selective medium for at least 1 week. Control experiments using either sonicated mitochondria or without the addition of ρ^+^ mitochondria failed to produce OxPhos competent clones, suggesting that intact organelles are required to render the recipient cells ρ^+^ (Table 1). Finally, we confirmed that the OxPhos competent clones contain recipient HOS cell nuclear DNA and the donor HEK293T cell mtDNA, by subjecting clones to SNP analysis (Fig. 1B,C). These data suggest that incubation of ρ^+^ mitochondria with ρ^0^ HOS cells leads to stable restoration of OxPhos function, consistent with assimilation of donor mitochondria by recipient HOS cells. This finding is also consistent with previously published data for other cell types^16,20^.

**Table 1:** Number of OxPhos competent clones produced in coincubation experiments.

		No mitochondria	Sonicated mitochondria	Isolated mitochondria
Number of OxPhos competent clones*	Exp. 1	0	0	8
	Exp. 2	0	0	10
	Exp. 3	0	0	11
	Average	0	0	9.67

^*^Number of clones is calculated per 1.5 × 10^5^ HOS ρ^0^ cells using 125 µg/ml of isolated mitochondria.

### Compounds that interfere with macropinocytosis block internalisation of mitochondria by HOS cells

In order to investigate if endocytic pathways were responsible for mitochondrial entry into HOS cells, we used chemical inhibitors of different pathways to determine if these compounds were able to prevent mitochondrial internalisation by cells. Firstly, drug concentrations were optimised in HOS cells using appropriate fluorescent cargo molecules and three different endocytic pathway inhibitors. Internalisation of fluorescent molecules by HOS cells was tested using FACS. We analysed the following marker/inhibitor pairs: Alexafluor-transferrin**/**dynasore, FITC-CTB/chlorpromazine (CPZ), FITC-CTB**/**MβCD, FITC-dextran**/**EIPA and FITC-dextran/wortmannin (Fig. S1)^21–23^. Transferrin and CTB are commonly used to monitor internalisation by CME and caveolae-mediated endocytosis respectively. 70 kDa dextran and other high molecular weight dextrans are preferentially internalised by macropinocytosis and they are routinely used to study internalisation by this endocytic pathway^24,25^. The data obtained suggested that HOS cells operate various endocytic pathways and helped to establish working concentrations for the inhibitory compounds (Fig. S1).

**Figure 2:**
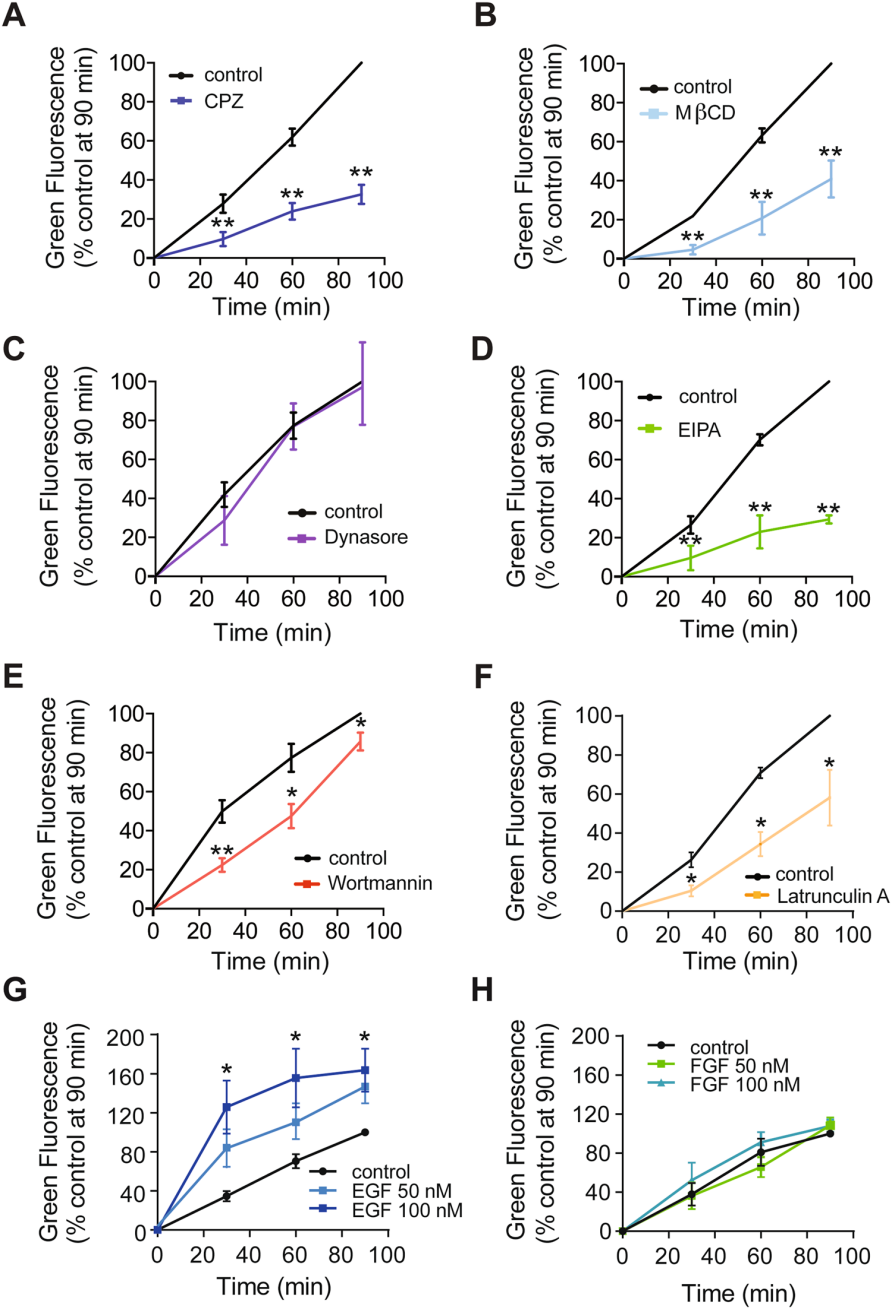
Internalisation of mitochondria occurs by endocytosis in HOS cells, with a macropinocytic activator EGF upregulating the process. (**A**–**F**) Internalisation of EGFP-labelled mitochondria in HOS cells, as quantified by FACS, in the presence and absence of the endocytic inhibitors: chlorpromazine (CPZ) (100 μm) (**A**), MβCD (5 mM) (**B**), dynasore (120 μm) (**C**), EIPA (50 μm) (**D**), wortmannin (300 nM) (**E**) and latrunculin A (0.5 µM) (**F**). (**G**,**H**) EGF upregulates mitochondrial internalisation. Internalisation of EGFP-labelled mitochondria by HOS cells, as quantified by FACS, in the presence and absence of 50 nM and 100 nM EGF (**G**), 50 nM FGF and 100 nM FGF (**H**). The Y axis shows the percentage of green fluorescent cells normalised to the numbers of green cells in a control sample (cells incubated with only EGFP-labelled mitochondria for 90 min). The number of green fluorescent cells in this control sample was taken to represent 100%. The green fluorescence in all samples for inhibitor assays of mitochondrial internalisation were normalised to levels in their respective control samples. Data shown as mean values +/− s.e.m. n = 3 (**A**–**D** and **F**–**H**) n = 6 (**E**). *p < 0.05 **p < 0.01.

We then employed the same FACS-based assay to quantify the number of HOS cells containing EGFP-labelled mitochondria in the presence and absence of endocytic pathway inhibitors. Test HOS cells were pre-treated with inhibitors followed by incubation with mitochondria labelled with nuclearly-encoded, mitochondrially-targeted EGFP, which were isolated from HEK293T cells. Cells were treated with trypsin, washed, and then analysed for green fluorescence by FACS to quantify the proportion of EGFP-positive cells. Both CPZ and MβCD impaired mitochondrial entry into cells (Fig. 2A,B). These drugs are classically used to inhibit clathrin-mediated endocytosis (CME) and caveolae-mediated endocytosis. However, they also affect macropinocytosis. MβCD extracts cholesterol from the plasma membrane, impairing membrane ruffle formation and CPZ inhibits phospholipase C (PLC), which acts in macropinosome closure^12,26,27^. The dynamin inhibitor dynasore is used to block CME and caveolae-mediated endocytosis^21^. There is evidence that dynasore also inhibits circular dorsal ruffling, which is associated with macropinocytosis^28^. However, it is not known to inhibit lamellipodial or bleb-associated macropinocytosis and dynasore did not prevent mitochondrial internalisation by cells (Fig. 2C). Moreover, mitochondrial entry was impaired in the presence of compounds that inhibit macropinocytosis (Fig. 2D–F). EIPA blocks the Na^+^/H^+^ transporter, leading to acidification of sub-membranous pH and disruption of the macropinocytic signalling cascade. Macropinocytosis is much more sensitive to the effects of acidification than CME^29^. Wortmannin inhibits PI3K, which is required for macropinosome closure^30^ and latrunculin A binds monomeric actin, preventing its incorporation into filaments, which results in disruption to actin polymerisation and macropinocytosis^31,32^. The overall pattern of inhibition provided by these three compounds (EIPA, wortmannin and latrunculin A), is consistent with effects on an actin-dependent pathway, possibly macropinocytosis. Two other studies have suggested that macropinocytic inhibitors interfere with mitochondrial uptake into human cells^16,18^.

### Mitochondrial entry is enhanced by stimulating macropinocytosis

Macropinocytosis is known to be stimulated by ligand binding to growth factor receptor tyrosine kinases, resulting in the activation of a signalling cascade, which regulates actin dynamics and macropinosome closure. Epidermal growth factor (EGF) enhances macropinocytosis in epithelial cell lines^33,34^. Therefore, if mitochondria enter HOS cells by macropinocytosis, stimulating cells with EGF should cause an increase in mitochondrial internalisation. In order to examine the effect of growth factors on mitochondrial internalisation, cells were incubated with EGFP-labelled mitochondria in the presence of either EGF or fibroblast growth factor (FGF). Samples were analysed by FACS to quantify the numbers of cells containing EGFP-labelled mitochondria. Treatment with EGF during mitochondrial uptake assays caused a 1.5 fold increase in mitochondrial internalisation by cells (Fig. 2G), whereas treatment with FGF, which affects cell migration and proliferation but not macropinocytosis, did not affect mitochondrial entry (Fig. 2H)^35^.

### Internalised mitochondria are transported in vesicles

**Figure 3:**
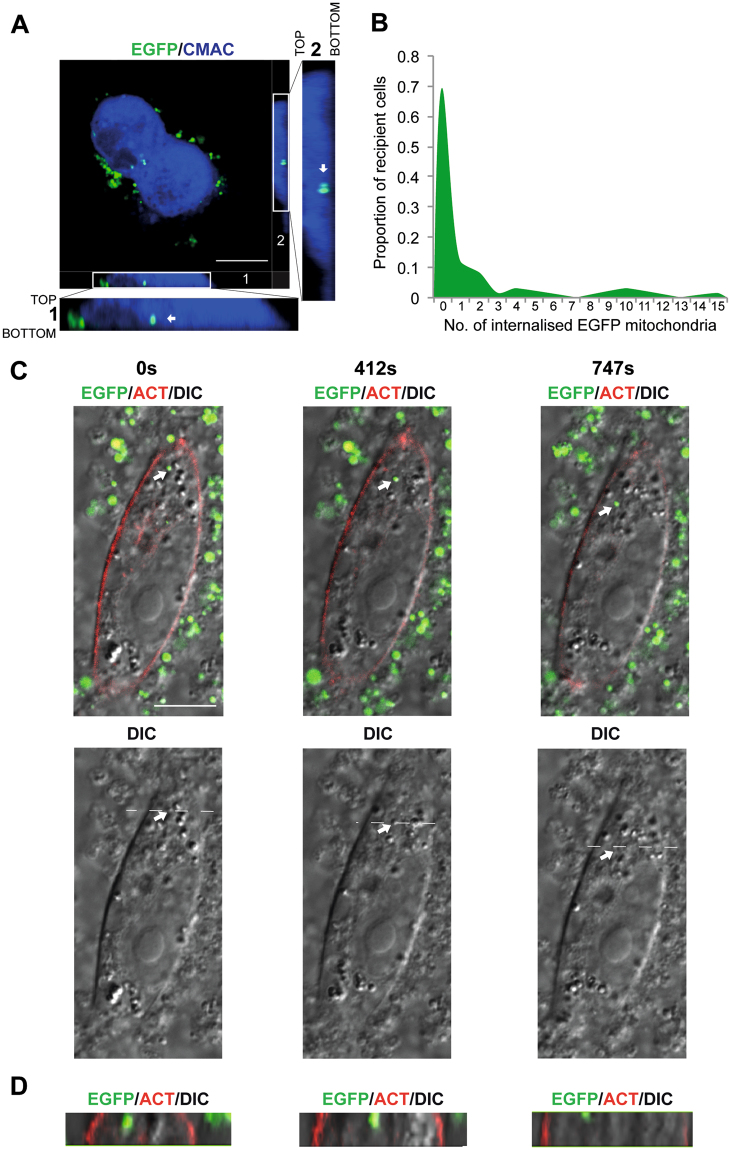
HOS cells internalise mitochondria in large vesicles. (**A**) HOS cells were incubated with EGFP-labelled mitochondria for 90 min and stained with a cytoplasmic dye (CMAC). Anti-GFP antibodies with Alexa Fluor 488 secondary antibodies were used to detect signal from EGFP-labelled mitochondria. Merged slice view of HOS cell after mitochondrial uptake. Full thickness sections through the z axis are shown in the boxed areas and these are enlarged in views 1 and 2. Views 1 and 2 show EGFP-labelled mitochondria indicated by arrows within a HOS cell. Scale bar 10 μm. (**B**) Proportion of HOS cells containing EGFP-labelled mitochondria identified by confocal microscopy in z axis sections. (**C**) Mitochondrial internalisation in live cells. HOS cells transfected with p^CMV^ Lifeact-TagRFP were incubated with EGFP-labelled mitochondria for 90 min at 37 °C. Time-lapse images of a HOS cell containing an EGFP-labelled mitochondrion. Arrow indicates an EGFP-labelled mitochondrion within a motile vesicle. DIC view showing vesicular structure (arrow). (**D**) Z axis view through HOS cell showing intracellular EGFP-labelled mitochondrion. Position of the section is indicated by dashed lines in the DIC image. Scale bar 10 μm.

The growth factor stimulation and inhibitor assays, suggested that mitochondria enter cells by macropinocytosis. In order to show this process in action we imaged mitochondrial uptake in cells using confocal microscopy. Firstly, we demonstrated that EGFP-labelled mitochondria were localised inside cells by using a cytoplasmic stain to delineate internal cell volumes (Fig. 3A). Cells samples produced for microscopy analysis could not be treated with trypsin and this is a likely explanation for the presence of extracellular EGFP-labelled mitochondria on cell surfaces. Furthermore, samples of EGFP positive cells identified by FACS were later examined by confocal microscopy to confirm whether the EGFP signal in these sorted cells originated from inside the cell or from the cell surface (Fig. S2). After 90 min of incubation 30% of cells contained EGFP-labelled mitochondria (Fig. 3B), which was consistent with the FACS-based analyses (Fig. 2). Next, we followed internalisation of EGFP-labelled mitochondria into HOS cells using time-lapse microscopy. HOS cells were transfected with an F-actin probe, Lifeact^36^ to define cell borders and then incubated with EGFP-labelled mitochondria. A 3 μm section through each cell was imaged in order to track internalised EGFP-labelled mitochondria. These studies showed that EGFP-labelled mitochondria are transported around cells in large, motile vesicles (0.5–1.5 μm in diameter) (Fig. 3C–D, Movie S1). It is theoretically possible that the intracellular vesicles observed in recipient cells are in fact remnants of cellular debris from mitochondria isolation. However, this seems very unlikely as such mitochondria containing vesicles would need to be present and remain stable in the hypotonic buffer used during mitochondria isolation. These debris derived vesicles would also need to be present in high quantities to be consistently observed inside cells during live mitochondrial uptake assays and to be responsible for the internalised mitochondria seen in fixed samples. Lastly, during any internalisation process these vesicles would acquire a second plasma membrane coat, producing double membraned structures. Double membraned vesicles containing mitochondria were not observed during our live assays (Fig. 3C–D). Taken together these data are consistent with the macropinocytic pathway of mitochondrial entry.

### Mitochondria are internalised with a known macropinocytic cargo molecule

Fluorescent cargo molecules known to be ingested by a particular endocytic pathway can be used to follow internalisation by that route^9^. Therefore, we used confocal time-lapse microscopy to confirm that HOS cells can macropinocytose, by adding 70 kDa FITC-dextran to cells and following its internalisation over time. The assays showed uptake of FITC-dextran into large, motile vesicles at the cell periphery, which were later transported more centrally within the cell (Fig. S2). These characteristics are consistent with macropinocytosis.

**Figure 4:**
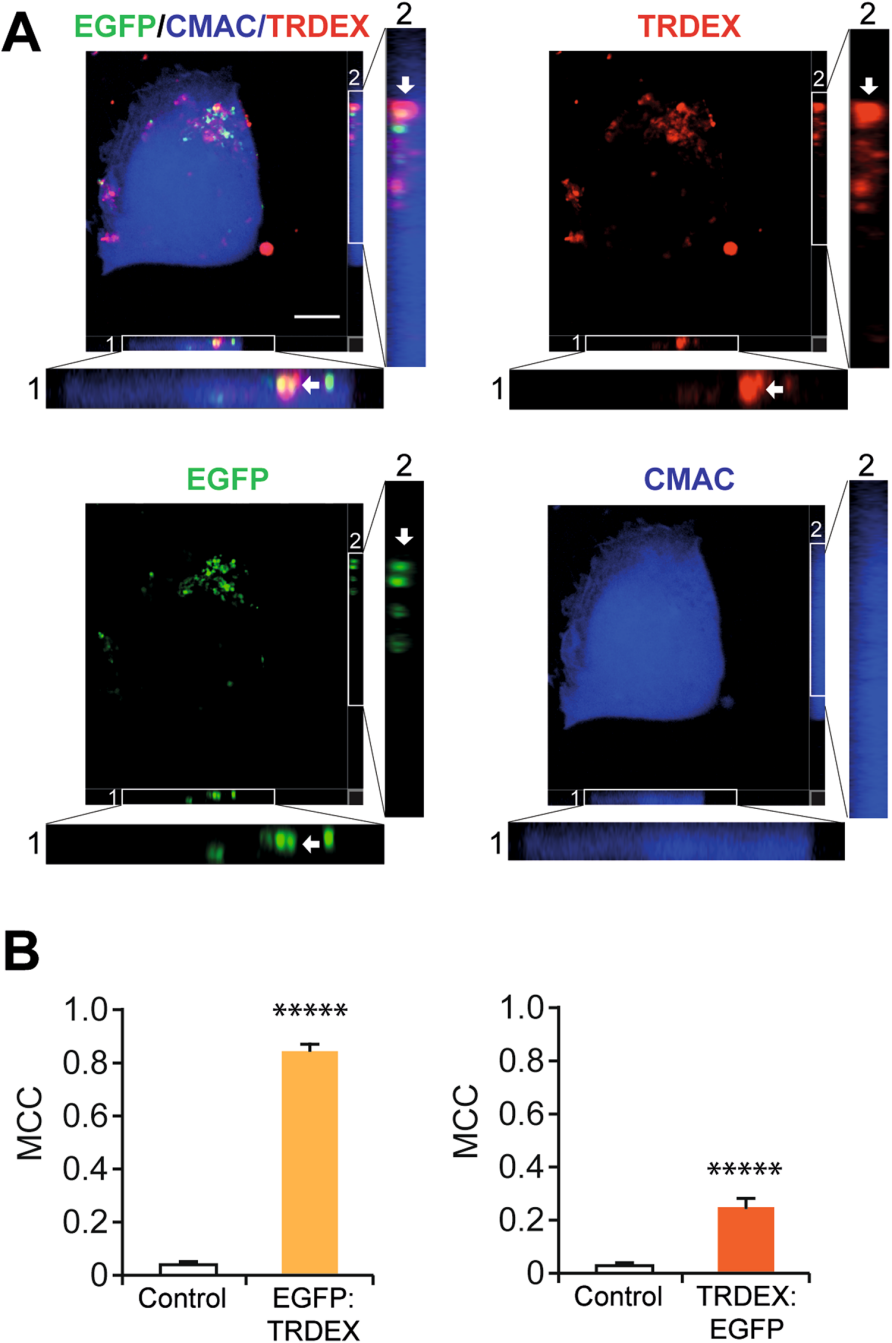
EGFP-labelled mitochondria colocalise with 70 kDa Texas Red dextran. (**A**) HOS cells were incubated with both EGFP-labelled mitochondria and 70 kDa Texas Red dextran (TRDEX), then stained with CMAC. Fields 1 and 2 are magnified views of z sections, arrows indicate EGFP-labelled mitochondria colocalised with Texas-Red dextran in the cell. (**B**) Quantification of colocalisation between EGFP-labelled mitochondria and Texas Red-dextran in images. Mander’s colocalisation coefficients (MCC) were calculated for EGFP and Texas Red-dextran in cells, thereby quantifying the fraction of EGFP colocalising with total intracellular Texas Red-dextran (M1) and the fraction of Texas Red-dextran colocalising with total intracellular EGFP (M2)^54^. Data shown as mean values +/− s.e.m. n = 16 *****p < 0.00001. 64 cells were imaged for the colocalisation assays.

Moreover, colocalisation of a cargo of interest with the fluorescent marker during internalisation suggests that they both enter by the same endocytic route. This approach is commonly used to investigate the endocytosis of routine cargo and pathogens^12^. High molecular weight dextran enters cells mainly by macropinocytosis. In order to investigate if mitochondria enter HOS cells by macropinocytosis, cells were analysed for colocalisation between EGFP-labelled mitochondria and 70 kDa Texas Red-dextran during internalisation assays using microscopy. EGFP-labelled mitochondria were found to colocalise with Texas Red-dextran during internalisation into cells (Fig. 4A). After coincubating 70 kDa Texas Red-dextran with EGFP-labelled mitochondria for 10 min, slice views through a HOS cell showed colocalisation of two separate EGFP-labelled mitochondria with a globular collection of Texas Red-dextran (Fig. 4A). Furthermore, quantification of colocalisation between EGFP-labelled mitochondria and Texas Red dextran in confocal images indicated highly overlapping signals (Fig. 4B). This suggests that EGFP-labelled mitochondria and 70 kDa dextran are internalised by the same endocytic route, again pointing at macropinocytosis as the mitochondrial uptake pathway.

### Confocal imaging shows that isolated mitochondria are associated with cellular structures characteristic for macropinocytosis

**Figure 5:**
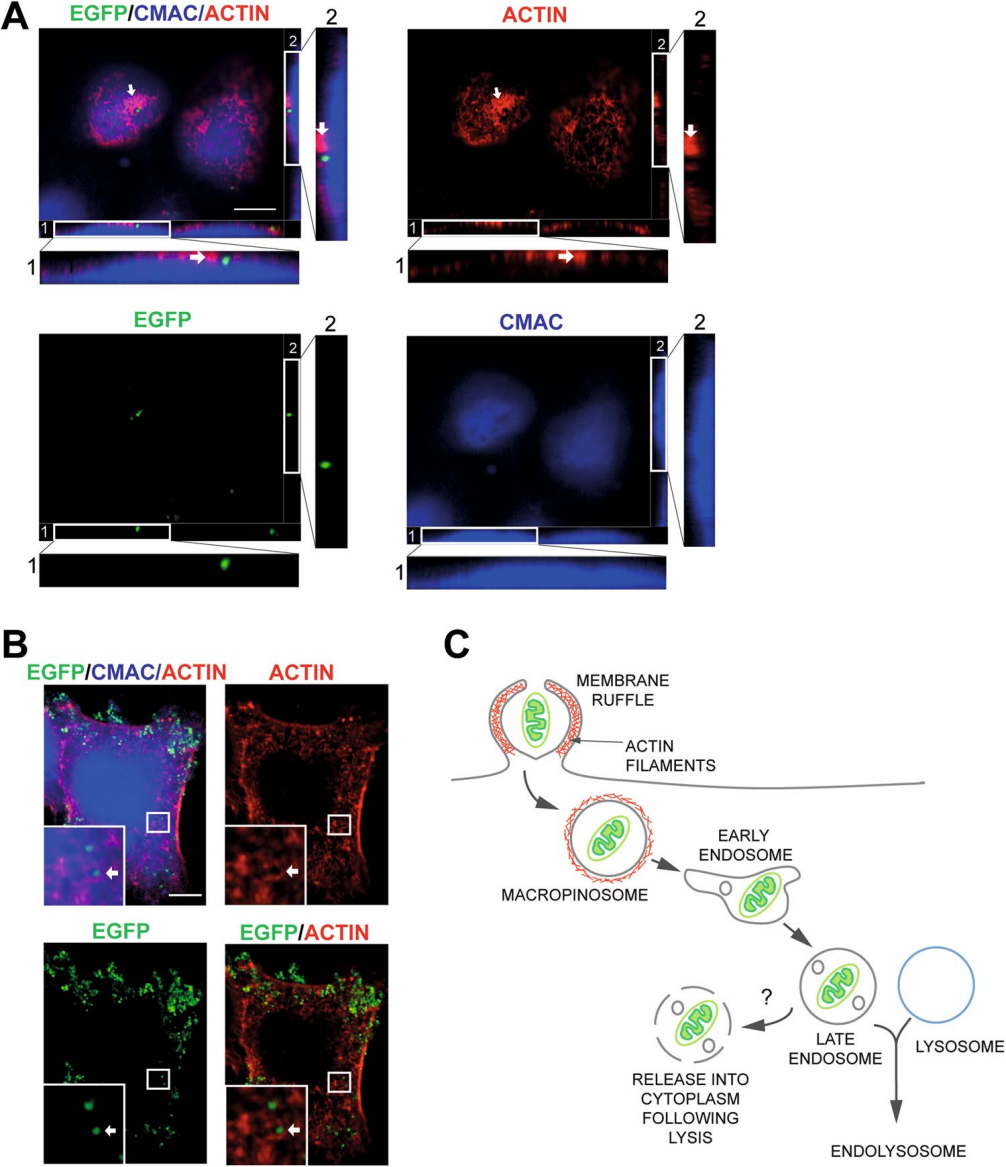
Isolated mitochondria are internalised by macropinocytosis in HOS cells. (**A**) A representative image of a HOS cell incubated with EGFP-labelled mitochondria and 100 nM EGF added 10 min before the end of the assay. Cells were stained with CMAC and Alexafluor 568 phalloidin (indicated as ACTIN). Fields 1 and 2 are magnified views of z sections, arrows indicate an actin-rich, semicircular-shaped membrane ruffle extending around an EGFP-labelled mitochondrion on the cell surface. (**B**) A representative image of an EGF-stimulated (100 nM, 10 min) HOS cell incubated with EGFP-labelled mitochondria as described in (**A**). Cells were stained Alexafluor 568 phalloidin (ACTIN). Insets are magnified views, with arrows indicating an EGFP-labelled mitochondrion inside a macropinosome coated with F-actin. Mitochondria were detected within early macropinosomes in 7 out of 30 imaged cells. Scale bar 10 µm. (**C**) Model of mitochondrial entry in HOS cells showing potential downstream trafficking events. After entering the cell in macropinosomes, internalised mitochondria are likely to join the endosomal system. Early endosomes mature into late endosomes. These can fuse with lysosomes to produce endolysosomes, leading to degradation of their contents. Some internalised mitochondria must escape the endosomal compartment in order to return to the cytoplasm. This might occur by lysis of the endosome, but at the moment the mechanisms remain unknown.

Finally, imaging studies were carried out during internalisation assays, which were capable of showing features specific to macropinocytosis. We examined whether mitochondria are associated with membrane ruffles and macropinosomes, since these structures are characteristic of macropinocytosis. We used confocal microscopy to analyse HOS cells incubated with EGFP-labelled mitochondria and 100 nM EGF, followed by staining with phalloidin for F-actin, as membrane ruffles are F-actin rich. These cells had ridge-like membrane ruffles on their dorsal surfaces, which were engulfing EGFP-labelled mitochondria (Fig. 5A).

Macropinosomes are large vesicles formed by the closure of membrane ruffles at the cell surface. Newly formed macropinosomes are transiently associated with a circular coat of F-actin. This feature of early macropinosomes is commonly used to identify them^37^. To determine if internalised mitochondria are found within macropinosomes, we used confocal microscopy, examining HOS cells incubated with EGFP-labelled mitochondria and 100 nM EGF. We observed EGFP-labelled mitochondria inside large vesicles (~0.8 μm in diameter), which were surrounded by F-actin arranged into a circular configuration (Fig. 5B). These results provide further evidence that exogenous mitochondria are internalised by macropinocytosis.

## Discussion

Multiple endocytic pathways operate within cells and it is necessary to analyse all known features for an endocytic pathway when investigating if a cargo is internalised by a particular route. Collectively these different lines of evidence can robustly indicate that the entry of a cargo is mediated by a particular pathway.

We have shown that isolated mitochondria enter HOS cells by macropinocytosis using a number of different approaches including endocytic inhibitors, macropinocytic agonists, colocalisation assays with known macropinocytic cargo molecules and imaging to demonstrate labelled mitochondria both interacting with membrane ruffles and also enclosed within macropinosomes. These independent lines of evidence all indicate that mitochondria enter HOS cells by macropinocytosis.

However, it is possible that mitochondria enter HOS cells by another pathway called phagocytosis, which internalises large extracellular particles. Phagocytosis uses pseudopod plasma membrane extensions to engage with cargo. Multiple direct receptor-mediated interactions are required between the pseudopods and an opsonised particle (opsonins are molecules which bind to antigen surfaces marking them as targets for the immune response) for successful internalisation. Moreover, specialised receptors FcRs (usually found on cells of the immune system) or CR3 complement receptors are required to mediate the interaction between the phagocytic cell and the particle surface^38-40^. We studied mitochondrial internalisation in human osteosarcoma cells. As non-immune system cells, they are unlikely to be able to phagocytose since they lack both FcRs and sources of opsonins (such as immunoglobulin or complement) required to activate this pathway. In addition, our data show that mitochondrial entry in HOS cells is enhanced by EGF, which is consistent with internalisation occurring by macropinocytosis. There is no evidence that EGF is able to upregulate phagocytosis in non-immune system cells.

We have also shown that endocytic transfer can be used to stably repopulate ρ^0^ HOS cells with intact ρ^+^ mitochondria. Therefore, macropinocytosis can be used to deliver mitochondria to cells in order to alter their mitochondrial content. Importantly, in this study we have shown that mitochondrial internalisation can be enhanced with EGF, a macropinocytic stimulator. Our data indicate that stimulating the macropinocytic pathway is able to promote mitochondrial entry in HOS cells. Perhaps, upregulating macropinocytic signalling, not necessarily by EGF, might be considered as an approach for improving the efficiency of mitochondrial internalisation by cells. This could be applied in studies using isolated mitochondria as therapeutic agents.

The main aim of using EGF in some of our experiments was to enhance underlying levels of macropinocytosis and make it easier to capture features and structures pertaining to this process during mitochondrial internalisation assays. Other studies have used EGF mediated stimulation of macropinocytosis to show that cargo such as exosomes are internalised by this pathway^33,34^. If mitochondria were not internalised by this process, these macropinocytic characteristics would be unlikely to be detected in association with mitochondria, even in the presence of EGF.

Although we have provided robust evidence for the mechanism of mitochondrial entry into HOS cells, it is unclear if other human cell types are able to internalise mitochondria using the same mechanism. Also, it is uncertain how these mitochondria are incorporated into the recipient cell mitochondrial population. After internalisation, mitochondria must escape the endocytic compartment in order to re-enter the cytoplasm. It is unlikely that they they perform OxPhos within the acidic environment of an endosome. Our data and that of others^13,18,20^ showing that imported mitochondria are both functional and stably maintained in recipient cells suggests that mitochondria can escape the endomembrane system without being degraded. However, the mechanisms of this process remain unknown. Internalised material from most endocytic pathways are routed into the endosomal pathway^41^. This membrane bound compartment is involved in the sorting, processing and recycling of endocytosed cargo^42^. There is evidence that pathogens entering cells by endocytosis escape from the endosomal compartment into the cytoplasm. For example, vesicular stomatitis virus (VSV) is released into the cytoplasm from late endosomes by back-fusion of endosomal carrier vesicles containing virus with the endosomal membrane^43^. Therefore, it is possible that internalised mitochondria are also returned to the cytoplasmic compartment, allowing them to join the recipient cell mitochondrial network (Fig. 5C). Further work might involve using imaging techniques to map the route of internalised mitochondria through the endosomal pathway and determine their destinations in the cell.

Studying the processes involved in mitochondrial internalisation has become more relevant, with interest in using mitochondria as therapy for diseases such as acute lung injury, heart attacks and stroke^5,8,44^. Recent evidence also suggests that intercellular mitochondrial transfer promotes tumour growth *in vivo* ^45^. Strategies for interfering with this process could provide novel therapeutic interventions against cancers. Although extensive work is necessary to fully investigate the therapeutic benefits of such treatments, methods of enhancing mitochondrial internalisation by target cells may be important in optimising mitochondrial therapies.

Several different approaches for delivering mitochondria to mammalian cells exist. These include cybrid generation^46^, intracellular microinjection of mitochondria^47^, creating transient pores within cell plasma membranes using a photothermal nanoblade^20^ and using a magnetic field to move mitochondria attached to magnetic beads into cells^48^. Of the currently available techniques, using macropinocytosis for mitochondrial transfer exploits a physiological process in many cell types and allows simultaneous delivery of mitochondria to multiple cells without introducing foreign material. Therefore, macropinocytosis may be better suited for delivering mitochondria to cells for *in vivo* applications. However, much additional work needs to be done to evaluate mitochondrial delivery approaches within *in vivo* models.

## Methods

### Materials

Chlorpromazine, MβCD, EIPA, latrunculin A, wortmannin, dynasore, FITC-dextran, FITC-CTB and Alexafluor 488 Transferrin were obtained from Sigma Aldrich (St. Louis, MO). DMEM, culture antibiotics, CellTracker^TM^ Blue, Alexa Fluor 568 phalloidin, and anti-GFP Ab from Life Technologies (Grand Island, NY). TaqGold DNA polymerase and BigDye™ Terminator (Applied Biosystems). Shrimp Alkaline Phosphatase and exonuclease1 (New England Biolabs). Fetal bovine serum from HyClone Laboratories (Logan, UT). BD Cellfix from BD Biosciences (USA). Microslides from Ibidi.

### Cell lines and cell culture conditions

HOS ρ^+^ cells were grown in DMEM with 10% FCS, streptomycin (100 μg/ml) and penicillin (100 U/ml). HOS ρ^0^ and HEK293T ρ^0^ cells obtained by treatment of HOS cells with ethidium bromide in a standard, previously described method^49^. Also, these ρ^0^ cells were unable to grow in a medium lacking uridine or a medium containing galactose as a sole carbon source^50^. Both types of ρ^0^ cells were cultured in DMEM with 10% FCS, streptomycin (100 μg/ml), penicillin (100 U/ml) and uridine (50 μg/ml).

To allow mitochondrial localisation of enhanced GFP (EGFP), a sequence coding for a mitochondrial targeting sequence (MTS), from the human ATP5B gene (which encodes the F_1_β subunit of mitochondrial ATP synthase) was inserted in frame, at the 5′ end of the EGFP cDNA. The construct was cloned into the pcDNA5/FRT/TO vector, after the addition of compatible restriction sites *BamHI* and *EcoRV* using PCR. Mitochondrial localisation of MTS-EGFP was verified by immunofluorescence (Fig. S6). A HEK293T cell line with tetracycline inducible expression of mitochondrially targeted EGFP (HEK EGFP cells) was produced by co-transfecting HEK293T cells with pcDNA5/FRT/TO/MTS-EGFP and pOG44 and selecting for integration at the genomic FRT site. Expression of mitochondrially targeted EGFP by cells was induced using doxycycline (50 ng/ml) and this produced mitochondria that were labelled with EGFP. HEK293T EGFP cells were grown in DMEM with 10% Tet^-^ FCS, blastocidin (10 μg/ml) and hygromycin (50 μg/ml).

### Mitochondrial isolation

We have used the standard method for mitochondria isolation from cultured cells as described previously^51,52^. All mitochondrial isolation steps were performed on ice at 4 °C. HEK EGFP cells that had been induced with doxycycline 50 ng/ml were harvested and collected by centrifugation for 5 min at 400 g in a 5810R Eppendorf centrifuge. Cells were resuspended in hypotonic buffer (0.6 M mannitol, 10 mM Tris, 1 mM EDTA, 1 mM PMSF and 0.1% BSA). They were lysed in a 3 ml homogeniser with 15 strokes per sample and then centrifuged at 400 g for 10 min at 4 °C to remove debris. The supernatant was taken off, the remaining pellet resuspended in hypotonic buffer and re-homogenised. Supernatants from each successive spin were combined and spun at 400 g for 5 min to remove remaining debris. These supernatants were then spun at 11000 g for 10 min to pellet mitochondria. Pellets were resuspended in 100 μl of hypotonic buffer without BSA. The quantity of mitochondria isolated from HEK293T GFP cells was determined using a BCA protein assay. The enrichment of mitochondria in the isolated fraction was measured by western blotting (Fig. S7).

### Mitochondrial uptake assays

To select respiratory competent clones, the uptake assays were performed within an hour of mitochondrial isolation, with the mitochondrial fraction being kept at 4 °C in the isolation buffer before the procedure. Immediately before experiments, mitochondrial isolation buffer was removed from the pellet and mitochondria were resuspended in calcium free DMEM. HOS ρ^+^ cells were seeded at densities of 1.5 × 10^5^ cells/ml in 6 well plates and grown in 800 μl of medium per well with supplementation of neomycin (500 μg/ml). Assays were performed after 24 hrs in confluent wells. Mitochondria isolated from ρ^+^ HEK 293 T were added at a concentration of 125 µg/ml to medium overlying HOS ρ^0^ cells, incubated at 37 °C in humidified air with 5% CO_2_. for 90 min and then in calcium-free medium for 24 hrs. Later medium was replaced by a standard DMEM supplemented with uridine and pyruvate for a further 24 hrs. OxPhos competent HOS cells were selected in DMEM medium supplemented with pyruvate, neomycin and galactose, without uridine. Mitochondrial concentrations greater than 125 µg/ml did not result in a measurable increase of mitochondrial uptake efficiency.

For the FACS-based assays, HOS cells were pre-plated at 0.5 × 10^5^ in a 24 well plate. The medium was replaced with 150 µl of calcium free medium prior to commencing the assay. HOS cells were incubated with inhibitors for 30 min at 37 °C. The following concentrations of inhibitors were used: dynasore 120 μm, chlorpromazine (CPZ) 100 μm, MβCD 5 mM, EIPA 50 μm and wortmannin 300 nM. Next, 500 μg/ml µg of EGFP labelled mitochondria were then added to wells for 90, 60 and 30 min with HOS cells. Control wells did not contain any inhibitor. On completion of the assay cells were detached with trypsin-EDTA and washed in PBS to remove any mitochondria attached to cell surfaces. Cells were collected by centrifugation at 400 g for 3 min in a 5810R Eppendorf machine, then fixed in BD Cellfix and kept on ice.

### FACS-based uptake assays

HOS cells samples that had been incubated with mitochondria were analysed with a Fortessa flow cytometer. FACS data were analysed using FlowJo software. A hierarchical gating system was applied to data to identify green fluorescent cells in samples. The single cell population was determined for each sample. Then fluorescence plots of non-fluorescent and green control cells (containing green fluorescent marker molecule or EGFP tagged mitochondria) were used to create gates to classify cells as green fluorescent or non-fluorescent. The gating for defining green fluorescent HOS cells was then applied to sample cell populations. In each FACS experiment, 10,000 events were examined, resulting in the analysis of 3000–6000 single cells per sample.

### Immunofluorescence

HOS cells were grown on glass coverslips for 24 hrs. Mitochondrial uptake assays were carried out as described above. Cells were stained with CMAC cell tracker prior to fixing in 4% formaldehyde. Fixed cells were permeablised in 1% triton PBS and then blocked in a solution of 10% FCS in PBS (PBSS) for 1 hr. Cells were incubated with mouse anti-GFP antibodies at room temperature for 1.5 hrs and then washed three times in PBSS. Cells were then incubated with anti-mouse Alexa Fluor 488 for 1 hr. At this stage Alexa Fluor 568 phalloidin was added in some samples to stain F-actin. Slides were washed twice in PBSS and once in PBS. Slides were analysed by confocal microscopy (Nikon Ti microscope).

### Microscopic examination of EGFP positive cell population

After mitochondrial uptake assays, the EGFP positive cell population was sorted by FACS and seeded onto coverslips. Between 1000–2000 EGFP positive cells from each incubation sample of 30 min, 60 min and 90 min were seeded onto coverslips. Cells were allowed to attach overnight, stained with the cytoplasmic dye CMAC and fixed the next day. Cells were examined by confocal microscopy as described above to establish whether the EGFP signal in this sorted population came from inside the cell or from the cell surface.

### Time-lapse microscopy

HOS cells were grown for 24 hrs in microslide wells. Cells were transfected overnight with pLifeAct-TagRFP using Lipofectamine 2000 according to the manufacturer’s instructions. Mitochondrial uptake assays were carried out in the transfected cells as described previously. Cells were then transferred to a microscope incubator at 37 °C and imaged dually with DIC and fluorescence.

### Mitochondrial and nuclear genotyping

HEK293T, HOS cells and HOS cell clones after mitochondrial uptake were harvested and lysed. Cell lysates were treated with proteinase K and the total DNA was extracted using the protocol described by Reyes *et al*.^53^. For nuclear genotyping regions including the SNP sites rs7171171, rs478582 and rs3087243 were sequenced to identify the alleles present within each cell line. In short, PCR amplification was performed using TaqGold DNA polymerase as per manufacturer’s instructions. PCR products were treated with Shrimp Alkaline Phosphatase and exonuclease1. Sequencing reactions were performed using BigDye™ Terminator as per manufacturer’s instructions. Sequencing products were analysed using an ABI 3700 DNA Analyser (Perkin Elmer Applied Biosystems, Foster City, CA) and sequence traces analysed using the Staden package (http://staden.sourceforge.net/) to identify the alleles present at each SNP. The D-loop region of mt-DNA was amplified using PCR for mt-DNA genotyping. Primers:

ForD-loop: CGCAGTATCTGTCTTTGATTCCTGC

RevD-loop: ATTACACATGCAAGCATCCCCGTTC

Sanger sequencing was carried out by Beckmann-Coulter genomics.

### Statistical analysis

FACS data from mitochondrial uptake assays were compared using unpaired t-tests in Excel software. Values averaged from at least 3 independent experiments were compared. A P-value < 0.05 was considered significant.

## Electronic supplementary material

Supplementary Information

## References

[1] Wallace DC (1999). Mitochondrial diseases in man and mouse. Science.

[2] Taylor RW, Turnbull DM (2005). Mitochondrial DNA mutations in human disease. Nat Rev Genet.

[3] Spees JL, Olson SD, Whitney MJ (2006). Mitochondrial transfer between cells can rescue aerobic respiration. Proceedings of the National Academy of Sciences of the United States of America.

[4] Clark MA, Shay JW (1982). Mitochondrial transformation of mammalian cells. Nature.

[5] Islam MN (2012). Mitochondrial transfer from bone-marrow-derived stromal cells to pulmonary alveoli protects against acute lung injury. Nat Med.

[6] Ahmad T (2014). Miro1 regulates intercellular mitochondrial transport & enhances mesenchymal stem cell rescue efficacy. EMBO J.

[7] Davis CH (2014). Transcellular degradation of axonal mitochondria. Proc Natl Acad Sci USA.

[8] Hayakawa K (2016). Transfer of mitochondria from astrocytes to neurons after stroke. Nature.

[9] Doherty GJ, McMahon HT (2009). Mechanisms of endocytosis. Annu Rev Biochem.

[10] McMahon HT, Boucrot E (2011). Molecular mechanism and physiological functions of clathrin-mediated endocytosis. Nat Rev Mol Cell Bio.

[11] Swanson JA (2008). Shaping cups into phagosomes and macropinosomes. Nat Rev Mol Cell Biol.

[12] Mercer J, Helenius A (2009). Virus entry by macropinocytosis. Nat Cell Biol.

[13] Katrangi E (2007). Xenogenic transfer of isolated murine mitochondria into human rho0 cells can improve respiratory function. Rejuvenation Res.

[14] Yang YW, Koob MD (2012). Transferring isolated mitochondria into tissue culture cells. Nucleic Acids Res.

[15] Kitani T (2014). Direct human mitochondrial transfer: a novel concept based on the endosymbiotic theory. Transplant Proc.

[16] Kitani T, Kami D, Matoba S, Gojo S (2014). Internalization of isolated functional mitochondria: involvement of macropinocytosis. J Cell Mol Med.

[17] Pacak CA (2015). Actin-dependent mitochondrial internalization in cardiomyocytes: evidence for rescue of mitochondrial function. Biol Open.

[18] Kesner EE, Saada-Reich A, Lorberboum-Galski H (2016). Characteristics of Mitochondrial Transformation into Human Cells. Sci Rep.

[19] Ivanov AI (2008). Pharmacological inhibition of endocytic pathways: is it specific enough to be useful?. Methods Mol Biol.

[20] Wu TH (2016). Mitochondrial Transfer by Photothermal Nanoblade Restores Metabolite Profile in Mammalian Cells. Cell Metab.

[21] Macia E (2006). Dynasore, a cell-permeable inhibitor of dynamin. Dev Cell.

[22] Bourseau-Guilmain E, Griveau A, Benoit JP, Garcion E (2011). The importance of the stem cell marker prominin-1/CD133 in the uptake of transferrin and in iron metabolism in human colon cancer Caco-2 cells. PLoS One.

[23] Chao TY, Raines RT (2011). Mechanism of ribonuclease A endocytosis: analogies to cell-penetrating peptides. Biochemistry.

[24] Dharmawardhane S (2000). Regulation of macropinocytosis by p21-activated kinase-1. Mol Biol Cell.

[25] Khan AG (2010). Human rhinovirus 14 enters rhabdomyosarcoma cells expressing icam-1 by a clathrin-, caveolin-, and flotillin-independent pathway. J Virol.

[26] Grimmer S, van Deurs B, Sandvig K (2002). Membrane ruffling and macropinocytosis in A431 cells require cholesterol. J Cell Sci.

[27] Walenga RW, Opas EE, Feinstein MB (1981). Differential effects of calmodulin antagonists on phospholipases A2 and C in thrombin-stimulated platelets. J Biol Chem.

[28] Liu YW, Surka MC, Schroeter T, Lukiyanchuk V, Schmid SL (2008). Isoform and splice-variant specific functions of dynamin-2 revealed by analysis of conditional knock-out cells. Mol Biol Cell.

[29] Koivusalo M (2010). Amiloride inhibits macropinocytosis by lowering submembranous pH and preventing Rac1 and Cdc42 signaling. J Cell Biol.

[30] Araki N, Johnson MT, Swanson JA (1996). A role for phosphoinositide 3-kinase in the completion of macropinocytosis and phagocytosis by macrophages. J Cell Biol.

[31] Spector I, Shochet NR, Kashman Y, Groweiss A (1983). Latrunculins: novel marine toxins that disrupt microfilament organization in cultured cells. Science.

[32] Aleksandrowicz P (2011). Ebola virus enters host cells by macropinocytosis and clathrin-mediated endocytosis. J Infect Dis.

[33] Bryant DM (2007). EGF induces macropinocytosis and SNX1-modulated recycling of E-cadherin. J Cell Sci.

[34] Nakase I, Kobayashi NB, Takatani-Nakase T, Yoshida T (2015). Active macropinocytosis induction by stimulation of epidermal growth factor receptor and oncogenic Ras expression potentiates cellular uptake efficacy of exosomes. Sci Rep.

[35] Carter EP, Fearon AE, Grose RP (2015). Careless talk costs lives: fibroblast growth factor receptor signalling and the consequences of pathway malfunction. Trends Cell Biol.

[36] Riedl J (2008). Lifeact: a versatile marker to visualize F-actin. Nat Methods.

[37] Sun P (2003). Small GTPase Rah/Rab34 is associated with membrane ruffles and macropinosomes and promotes macropinosome formation. J Biol Chem.

[38] Ross GD, Reed W, Dalzell JG, Becker SE, Hogg N (1992). Macrophage cytoskeleton association with CR3 and CR4 regulates receptor mobility and phagocytosis of iC3b-opsonized erythrocytes. J Leukoc Biol.

[39] Anderson CL, Shen L, Eicher DM, Wewers MD, Gill JK (1990). Phagocytosis mediated by three distinct Fc gamma receptor classes on human leukocytes. J Exp Med.

[40] Griffin FM, Griffin JA, Leider JE, Silverstein SC (1975). Studies on the mechanism of phagocytosis. I. Requirements for circumferential attachment of particle-bound ligands to specific receptors on the macrophage plasma membrane. J Exp Med.

[41] Gruenberg J, van der Goot FG (2006). Mechanisms of pathogen entry through the endosomal compartments. Nat Rev Mol Cell Biol.

[42] Huotari J, Helenius A (2011). Endosome maturation. EMBO J.

[43] Le Blanc I (2005). Endosome-to-cytosol transport of viral nucleocapsids. Nat Cell Biol.

[44] Masuzawa A (2013). Transplantation of autologously derived mitochondria protects the heart from ischemia-reperfusion injury. Am J Physiol Heart Circ Physiol.

[45] Tan AS (2015). Mitochondrial genome acquistion restores respiratory function and tumourigenic potential of cancer cells without mitochondrial DNA. Cell Metabolism.

[46] King MP, Attardi G (1989). Human cells lacking mtDNA: repopulation with exogenous mitochondria by complementation. Science.

[47] King MP, Attardi G (1988). Injection of mitochondria into human cells leads to a rapid replacement of the endogenous mitochondrial DNA. Cell.

[48] Macheiner T (2016). Magnetomitotransfer: An efficient way for direct mitochondria transfer into cultured human cells. Sci Rep.

[49] King MP, Attardi G (1996). Isolation of human cell lines lacking mitochondrial DNA. Methods Enzymol.

[50] Gattermann N (2004). Severe impairment of nucleotide synthesis through inhibition of mitochondrial respiration. Nucleosides Nucleotides Nucleic Acids.

[51] Rorbach J (2014). MRM2 and MRM3 are involved in biogenesis of the large subunit of the mitochondrial ribosome. Mol Biol Cell.

[52] Van Haute L (2016). Deficient methylation and formylation of mt-tRNA(Met) wobble cytosine in a patient carrying mutations in NSUN3. Nat Commun.

[53] Reyes A, Yasukawa T, Holt IJ (2007). Analysis of replicating mitochondrial DNA by two-dimensional agarose gel electrophoresis. Methods Mol Biol.

[54] Dunn KW, Kamocka MM, McDonald JH (2011). A practical guide to evaluating colocalization in biological microscopy. Am J Physiol Cell Physiol.

