# Supplementary material for: Macropinocytic entry of isolated mitochondria in epidermal growth factor-activated human osteosarcoma cells
Source: Sci Rep. 2017 Oct 10;7:12886. doi: 10.1038/s41598-017-13227-0 (PMC5634993; doi:10.1038/s41598-017-13227-0)
Supplement: Supplementary file 1 — Supplementary Information [file 41598_2017_13227_MOESM1_ESM.doc]

**Supplementary Information**

**Macropinocytotic entry of isolated mitochondria in epidermal growth factor-activated human osteosarcoma cells**

# Dipali Patel, Joanna Rorbach, Kate Downes, Maciej Szukszto, Marcin L. Pekalski & Michal Minczuk


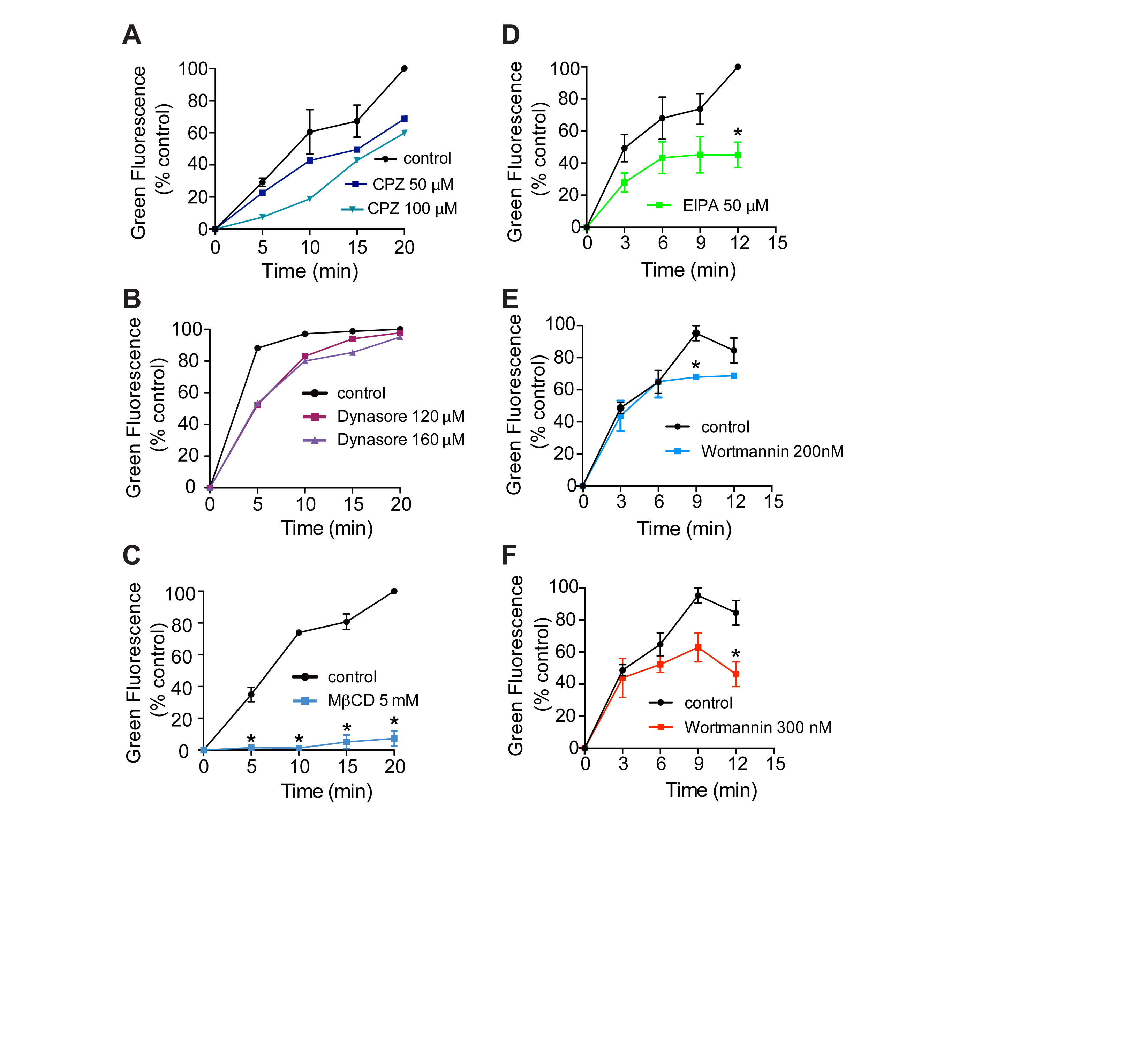
**Fig. S1 | HOS cells internalise cargo for CME, caveolae-mediated endocytosis and macropinocytosis**.

HOS cells were pre-incubated with inhibitors blocking different endocytic pathways. Cells were then incubated at 37ºC with 4 ng/ml FITC-CTxB, 50 mg/ml Alexa Fluor 488 Transferrin or 0.5 mg/ml FITC-dextran. Internalisation of FITC-CTxB/CPZ (A) or Alexafluor 488 Transferrin /dynasore (B). FITC-CTxB/MβCD (C). FITC-dextran/EIPA (D) or FITC-dextran/wortmannin (E and F). Samples were analysed by FACS to quantify the number of green fluorescent cells and values were normalised to control levels of green fluorescence. Data shown as mean values +/- s.e.m. * *p* < 0.05, n= 3 (C, D, E, F) n= 2 (A) n=1 (B).


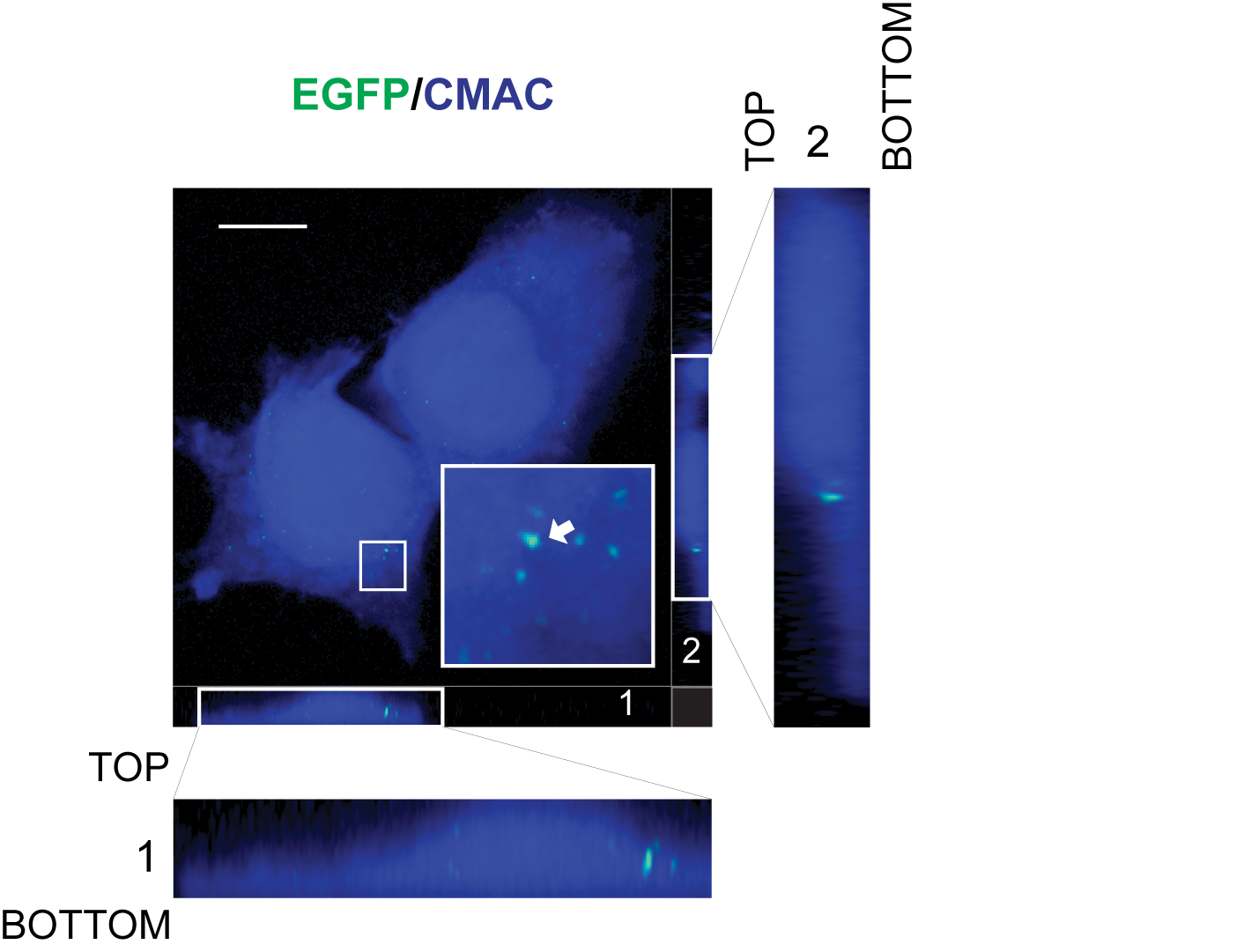


**Fig. S2 After mitochondrial uptake, FACS sorted EGFP-positive cells are confirmed to contain internalised EGFP.** Cells were incubated with EGFP-labelled mitochondria for 30 min, 60 min and 90 min. Samples were sorted by FACS into EGFP-positive populations, then plated onto coverslips, fixed and prepared for immunocytochemistry as described previously. Boxed areas in the image are enlarged as indicated. Merged view of HOS cells containing multiple punctate EGFP signals (see arrow in square inset) in the cytoplasm. Scale bar 10 µm. Image is representative of samples from three separate assays.

In order to assess if the FACS assay used to quantify mitochondrial uptake in cells was detecting internalised EGFP-labelled mitochondria rather than mitochondria attached to the cell surface, samples of EGFP-positive cells identified by FACS after mitochondrial uptake experiments were seeded onto coverslips and examined using confocal microscopy. In total

36 cells were imaged and all contained numerous, small, punctate EGFP signal, which were

scattered throughout the cytoplasm (see Fig. S2). A necessary delay between mitochondrial uptake, cell sorting, replating and fixing of samples allowed the cells more time to process internalised EGFP mitochondria. This is a likely reason for the distribution of EGFP signal in these images to be different from that in cells which were fixed immediately after incubation with mitochondria. Since the images show that the EGFP signal is intracellular, the data still suggest that the FACS-based method was able to correctly identify cells containing EGFP-labelled mitochondria and can be used to measure mitochondrial uptake.


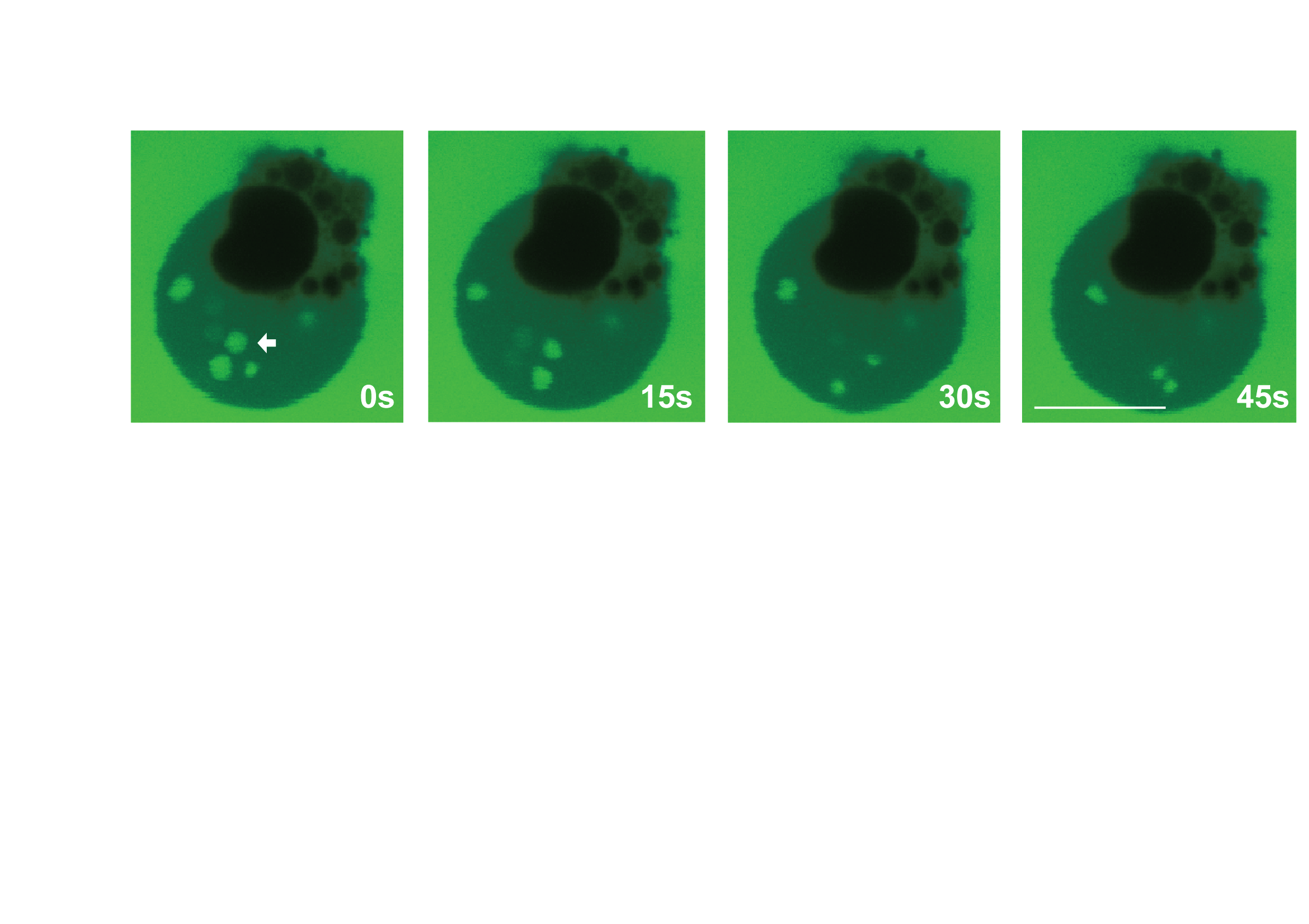


**Fig. S3** | **HOS cells internalise 70 kDa FITC-dextran into large, motile vesicles suggestive of macropinosomes.** FITC-dextran 0.5 mg/ml was added to cells immediately before assays. After a few minutes images of cells were acquired in a single focal plane every 15s for 10 min. FITC-dextran has been internalised by the HOS cell in large, vesicles 1-2 µm in diameter (arrow). These motile vesicles move away from the plane of focus during the time lapse. The first image shown in the time series was designated as t=0 and later time points are relative to this one (the actual time stamps of the images are not shown in “real” time). Scale bar 10 µm.


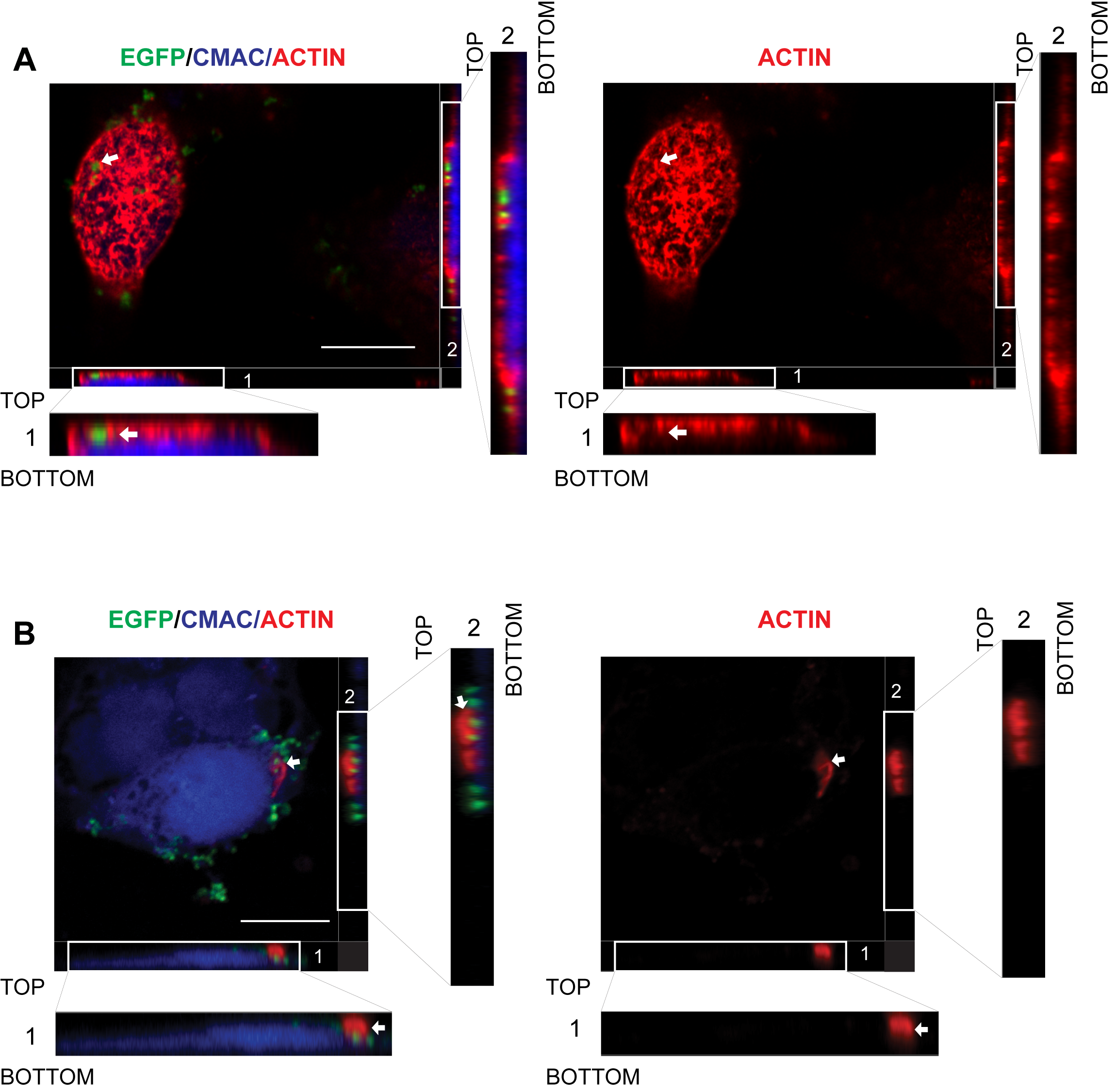


**Fig. S4 | Images of EGFP-labelled mitochondria associated with actin-rich membrane ruffles on HOS cell surfaces.**

**(A** and **B)** Cells were stained with CMAC and Alexa Fluor 568 phalloidin (ACTIN). Fields 1 and 2 are magnified views of z sections, arrows indicate ridge-like membrane ruffles extending around EGFP-labelled mitochondria. Scale bars 10 µm.


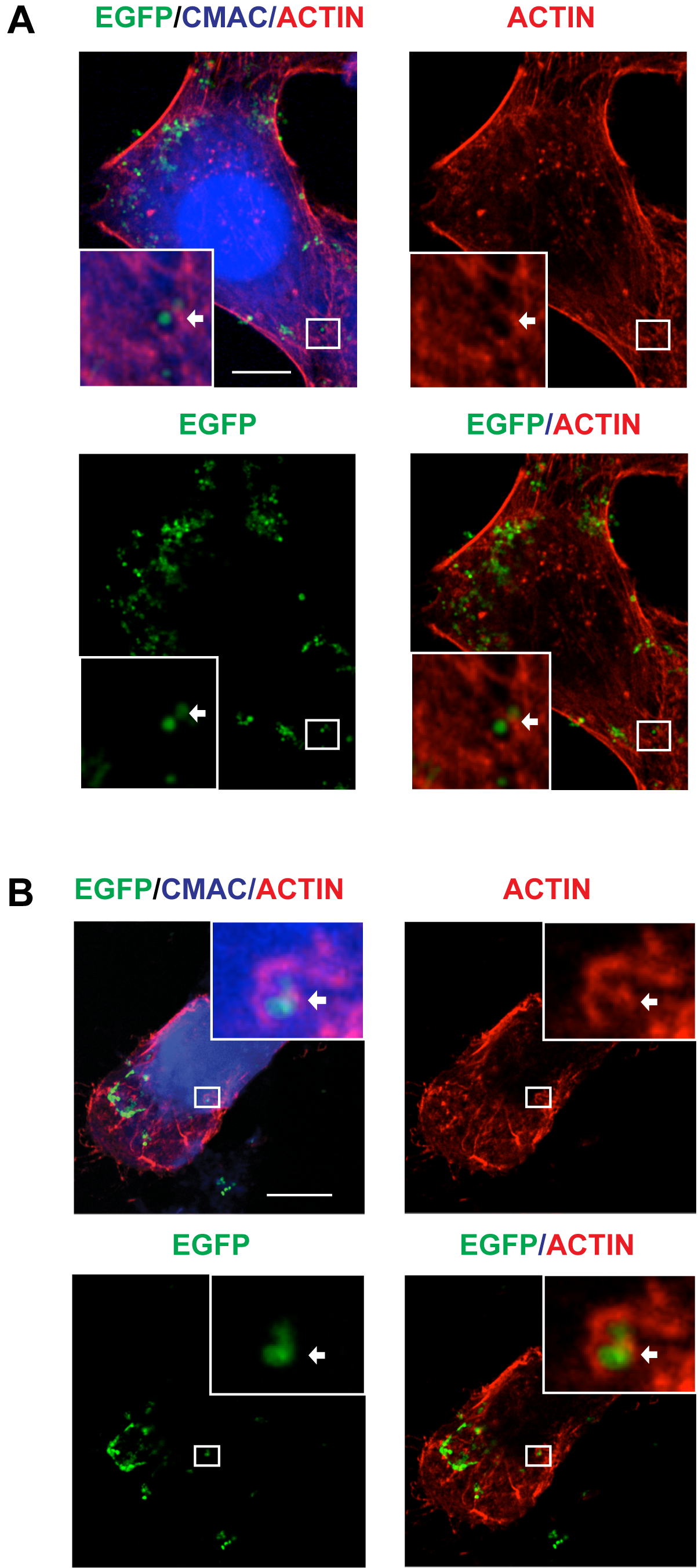


**Fig. S5 | Images of mitochondria within early macropinosomes.**

**(A** and **B)** Cells were stained with CMAC and Alexa Fluor 568 phalloidin (ACTIN). Insets are magnified views. Arrows indicate F-actin coated macropinosomes containing EGFP-labelled mitochondria. Scale bar 10 µm. Representative images are shown (7 images of macropinosomes containing mitochondria were obtained from a total of 30 cells).

**A B**

**
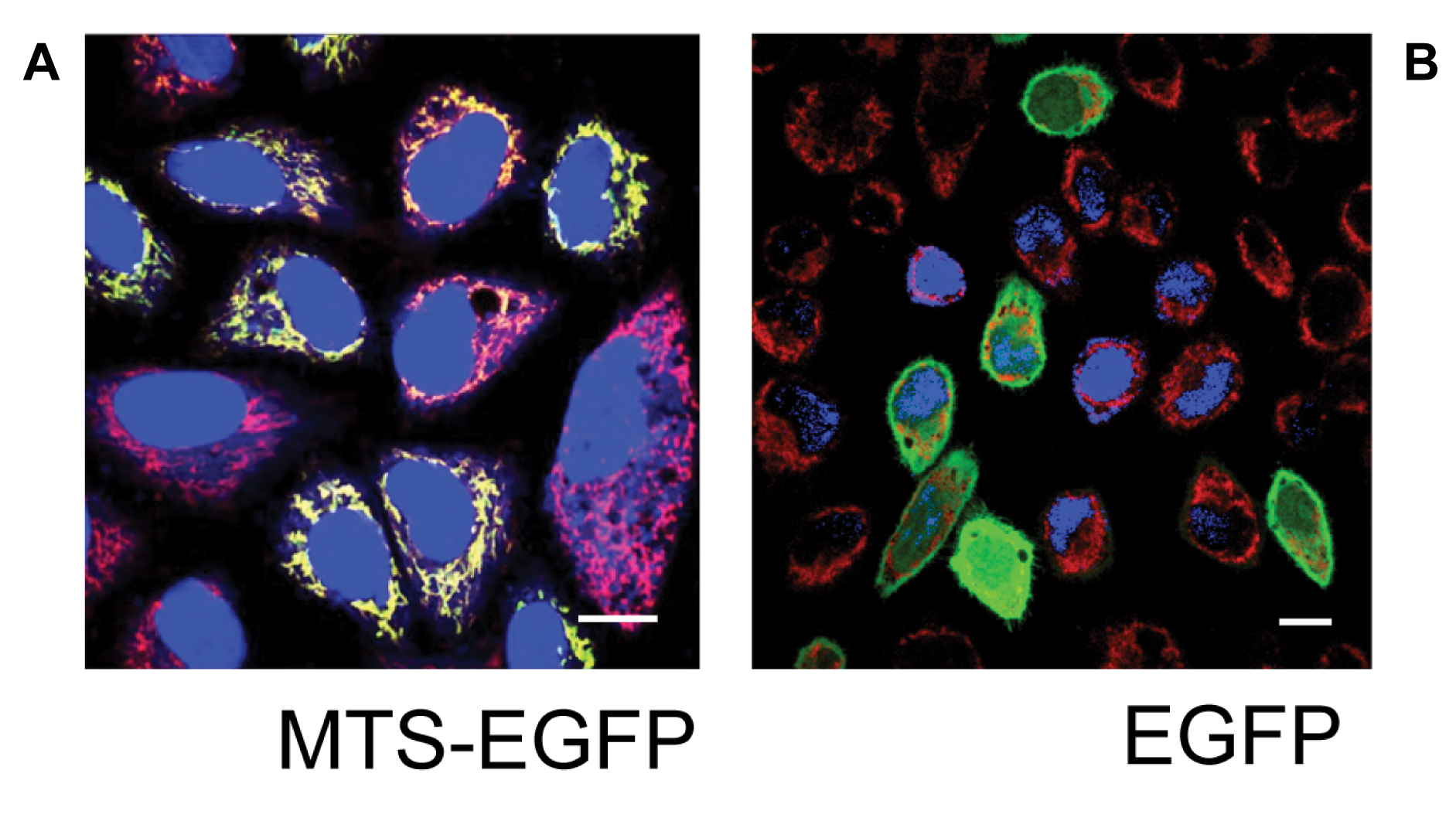
**

**Fig. S6 | Cellular localisation of mitochondrially targeted EGFP (MTS-EGFP) or untargeted EGFP control.**

The mitochondrial targeting sequence (MTS) was derived from the ATP5B gene, as described in Methods. Constructs expressing MTS-EGFP (A) or EGFP (B) were transiently transfected into HeLa cells. Mitochondria were immuno-stained with an antibody recognising TOM20 and Alexa Fluor 594 secondary antibody (red). Nuclei were stained blue with DAPI (blue). Colocalisation of EGFP with mitochondria appears yellow on digitally merged images. Scale bars 10 μm.

**Fig. S7 |** **Verification of mitochondrial enrichment upon cell fractionation.**

HEK293T cells were fractionated into cytosol (C, lane 2) and mitochondria (lanes 3-5). “T” (lane 1) indicates the total cell lysate. The fractions were analysed by western blotting using antibodies against marker proteins TOM22 (mitochondria) and GAPDH (cytosol). The purity of the mitochondrial fraction was assessed by analysing the GAPDH signal. The mitochondrial GAPDH signal was undetectable after the mitochondrial fraction was treated with 25 µg/ml proteinase K (lane 4) (Note: in these conditions TOM22 is truncated as observed previously e.g. Van Haute et al. Nat Commun. (2016) 30:12039). Disruption of membranes with 1% Triton X100 (lane 5) led to a complete proteolysis of the analyzed proteins.
